# Skin injuries due to Personal Protective Equipment and preventive measures in the COVID-19 context: an integrative review

**DOI:** 10.1590/1518-8345.5636.3522

**Published:** 2022-04-20

**Authors:** Lorrany Fontenele Moraes da Silva, Alana Gomes de Araujo Almeida, Lívia Maia Pascoal, Marcelino Santos, Francisca Elisângela Teixeira Lima, Floriacy Stabnow Santos

**Affiliations:** 1 Universidade Federal do Maranhão, Centro de Ciências Sociais, Saúde e Tecnologia, Imperatriz, MA, Brasil.; 2 Bolsista da Coordenação de Aperfeiçoamento de Pessoal de Nível Superior (CAPES), Brasil.; 3 Universidade Federal do Maranhão, Centro de Ciências Humanas, Naturais, Saúde e Tecnologia, Pinheiro, MA, Brasil.; 4 Universidade Federal do Ceará, Departamento de Enfermagem, Fortaleza, CE, Brasil.; 5 Bolsista do Conselho Nacional de Desenvolvimento Científico e Tecnológico (CNPq), Brasil.

**Keywords:** Personal Protective Equipment, Skin, Health Personnel, Skin Diseases, Disease Prevention, COVID-19, Equipamento de Proteção Individual, Pele, Profissionais da Saúde, Dermatopatias, Prevenção de Doenças, COVID-19, Equipo de Protección Personal, Piel, Personal de Salud, Enfermedades de la Piel, Prevención de Enfermedades, COVID-19

## Abstract

**Objective:**

to identify the diverse scientific evidence on the types of skin lesions caused due to the use of Personal Protective Equipment in health professionals during the COVID-19 pandemic and to verify the recommended prevention measures.

**Method:**

this is an integrative review carried out in the MEDLINE, CINAHL, LILACS, SCOPUS, Science Direct, Web of Science and SciELO databases. The search was conducted in a paired manner, constituting a sample of 17 studies categorized according to the types of skin lesions and preventive measures.

**Results:**

the main types of skin lesions related to mask use were stage 1 pressure ulcers, acne and cutaneous depression. Regarding the use of glasses and face shields, the most frequent were stage 1 and 2 pressure ulcers. Xerosis and irritant contact dermatitis occurred due to using gloves and protective clothing, respectively. The main preventive measures recommended were using hydrocolloid or foam dressing in the pressure regions, moisturizers and emollients.

**Conclusion:**

a considerable number of skin lesions associated with using the equipment were noticed, and the data obtained can guide the professionals in identifying risks and promoting preventive measures to avoid their occurrence.

## Resumo

**Objetivo:**

identificar as evidências científicas sobre os tipos de lesões de pele ocasionadas pelo uso de Equipamento de Proteção Individual em profissionais de saúde durante a pandemia da COVID-19 e verificar as medidas de prevenção recomendadas.

**Método:**

trata-se de uma revisão integrativa realizada nas bases de dados MEDLINE, CINAHL, LILACS, SCOPUS*, Science Direct*, *Web of Science* e no banco de dados SciELO. A busca foi realizada de forma pareada, constituindo uma amostra de 17 estudos categorizados quanto aos tipos de lesões de pele e às medidas preventivas.

**Resultados:**

os principais tipos de lesões de pele relacionados ao uso de máscara foram lesão por pressão estágio 1, acne e depressão cutânea. Quanto ao uso de óculos e protetor facial, as mais frequentes foram lesão por pressão estágios 1 e 2. Xerose e dermatites de contato irritante ocorreram devido ao uso de luvas e roupas de proteção, respectivamente. As principais medidas preventivas recomendadas foram o uso de curativo de hidrocoloide ou espuma nas regiões de pressão, hidratantes e emolientes.

**Conclusão:**

observou-se um número considerável de lesões de pele associadas ao uso dos equipamentos e os dados obtidos podem direcionar os profissionais na identificação de riscos e promoção de medidas preventivas para evitar sua ocorrência.

## Resumen

**Objetivo:**

identificar que evidencia científica hay sobre los tipos de lesiones cutáneas provocadas por el uso de Equipos de Protección Individual en los profesionales sanitarios durante la pandemia del COVID-19 y verificar las medidas de prevención recomendadas.

**Método:**

se trata de una revisión integradora realizada en las bases de datos MEDLINE, CINAHL, LILACS, SCOPUS, *Science Direct, Web of Science* y la biblioteca electrónica SciELO. La búsqueda se realizó de forma pareada, la muestra estuvo conformada por 17 estudios categorizados según los tipos de lesiones cutáneas y medidas preventivas.

**Resultados:**

los principales tipos de lesiones cutáneas relacionadas con el uso de mascarillas fueron las lesiones por presión estadio 1, el acné y la depresión cutánea. En cuanto al uso de gafas y pantalla facial, las más frecuentes fueron las lesiones por presión estadios 1 y 2. Se detectó xerosis y dermatitis irritante de contacto por el uso de guantes y ropa de protección, respectivamente. Las principales medidas preventivas recomendadas fueron el uso de apósitos de hidrocoloide o espuma en las regiones de presión, humectantes y emolientes.

**Conclusión:**

hubo un número considerable de lesiones cutáneas asociadas al uso de equipos y los datos obtenidos pueden orientar a los profesionales para que identifiquen los riesgos y promuevan medidas preventivas para evitar su aparición.

**Highlights**

(1) The main PPE items responsible for the occurrence of skin injuries are masks.

(2) Acne and stage 1 and 2 pressure ulcers are the main types of skin injury.

(3) The main skin injury prevention measures involve routine care measures.

(4) Health professionals usually do not adopt preventive measures when using PPE

## Introduction

In January 2020, COVID-19, a disease caused by the new Coronavirus (SARS-CoV-2), was considered an international public health emergency and, in March 2020, the World Health Organization (WHO) declared it as a pandemic^1^. Since then, health professionals have been dealing with many challenges, such as: prolonged exposure; work overload; lack of training; inadequate staffing; precariousness of the work environment; and Personal Protective Equipment (PPE) in insufficient quality and numbers, among other factors that jeopardize these professionals’ biosafety^2^. 

COVID-19 has stood out for its high transmissibility and clinical severity, and also for having a higher incidence among health professionals^3^. To reduce the risk of exposure in the professionals who are on the front lines in the fight against COVID-19, in its Technical Note No. 04/2020, the National Health Surveillance Agency (*Agência Nacional de Vigilância Sanitária*, ANVISA) recommends wearing PPE according to the standard precautionary type for droplets, aerosols and/or contact. Thus, to care for suspected or confirmed COVID-19 cases, goggles or face shield, cap, surgical or N95/PFF2 mask or equivalent (depending on the type of precaution), apron and sterile-procedure gloves for non-invasive procedures must be used^4^. 

Although PPE use is intended for protection, the professionals are also prone to the occurrence of skin lesions caused by their prolonged use and frequent hand hygiene, which can progress to pressure ulcers, acute and chronic dermatitis and worsening of preexisting dermatosis, as well as being a gateway to secondary infections^5^ ^-^ ^6^.

Prolonged contact with PPE affects the integrity of the skin barrier due to the force of continuous or sliding friction and the skin’s own structural condition, causing tissue deformation, which leads to the process of cell death and triggers tissue damage. It is also noteworthy that factors such as excessive sweating generated by mental stress and exhausting workdays and non-optimization of the PPE in the face of tissue deformation, in addition to the adaptation made by the professionals to improve sealing of the facial region, in search of overprotection, effectively contribute to tissue damage^7^. 

In this regard, it is important to highlight that tissue damage is a limiting factor for care, as health professionals, subjected to extensive working hours with physical and emotional exhaustion, have become a population at risk for the development of injuries and faced with the lack of adequate protective equipment, worsened signs and symptoms of skin lesions and/or pre-existing diseases and, in most cases, without access to prevention measures due to lack of knowledge and guidance as a result of scarcity of studies, the costs of such materials or even the lack of resources from hospital institutions^8^.

Considering that the protective equipment used in health care is indispensable, it becomes necessary to implement measures that help to preserve skin integrity in the area exposed to risk, measures that will directly contribute to protecting the skin and, consequently, to ensure care quality and patient safety. Therefore, the justification for this review is based on the contribution to the health professionals’ practice, especially nurses, for providing information about skin lesions associated with using PPE and the measures that can be adopted to prevent their occurrence because, in addition to favoring safe professional practice and promoting effective care for the assisted population, it will guide the professionals in similar situations that may occur, allowing for the development of risk anticipation strategies for the maintenance of skin integrity.

Therefore, the objective is to identify the diverse available scientific evidence on the types of skin lesions caused by PPE use in health professionals during the COVID-19 pandemic and to verify the recommended prevention measures.

## Method

### Type of study

This study consists of an integrative review filed on the FigShare platform^9^. The study was conducted in six stages: 1) definition of the research question; 2) definition of the databases and criteria for inclusion and exclusion of studies; 3) definition of the information to be extracted from the studies selected; 4) assessment of the studies included in the review; 5) interpretation of the results; and 6) presentation of the review/knowledge synthesis^10^. 

To guide the search, the research question was elaborated using the PICo (Population, Interest and Context) strategy: P - Health professionals; I - Types of injuries related to PPE use and Preventive measures; and Co - The COVID-19 Pandemic. This acronym allows reaching an effective search from the elaboration of an enlightening research question to direct the study according to the objectives proposed^11^. This strategy allowed formulating the following guiding question: “What types of skin lesions are caused by PPE use in health professionals during the COVID-19 pandemic and which are the recommended prevention measures?”.

An initial search was carried out in the Medical Literature Analysis and Retrieval System Online (MEDLINE) database, via the National Library of Medicine (PubMed), to verify the main descriptors or keywords used in the studies on the theme of the guiding question. Controlled vocabularies were selected from the Descriptors in Health Sciences (*Descritores em Ciências da Saúde*, DeCS), the Medical Subject Headings (MeSH) and MH Exact Subject Headings (CINAHL vocabulary), namely: *COVID-19*; *occupational injury*; *facial injuries*; *personal protective equipment*; *protective devices* and *skin.* As this is an incipient theme, it was decided to also use keywords (uncontrolled vocabularies) to obtain a targeted search strategy, namely: *device related pressure injuries, skin injury* and *skin damage*, used in the Spanish language for the LILACS and SciELO databases. To perform crossings among these words, the operators used were the Boolean operators *AND* and *OR* (Figure 1).

**Figure 1:** Search strategy for the studies according to the databases found. Imperatriz, MA, Brazil, 2021

Database	Crossing	Number
MEDLINE	*COVID-19 AND* (“*protective devices*” *OR* “*personal protective equipment*”) *AND* (*skin OR* “*device related pressure injuries*” *OR* “*facial injuries*” *OR* “*occupational injury*” *OR* “*skin injury*” *OR* “*skin damage*”).	99
CINAHL	(MH”*protective devices*”) *OR* (MH”*personal protective equipment*”) *AND* (*skin OR* “*device related pressure injuries*” *OR* (MH”*facial injuries*”) *OR* (MH “*occupational injury*”) *OR* “*skin injury*” *OR* “*skin damage*”)	45
LILACS	*COVID-19 AND* (“*dispositivos de protección*” *OR* “*equipo de protección personal*”) *AND* (*piel OR* “*lesiones por presión relacionadas con el dispositivo*” *OR* “*lesiones faciales*” *OR* “*lesión ocupacional*” *OR* “*lesión cutánea*” *OR* “*daño cutáneo*”)	1
SCOPUS	*COVID-19 AND* (“*protective devices*” *OR* “*personal protective equipment*”) *AND* (*skin OR* “*device related pressure injuries*” *OR* “*facial injuries*” *OR* “*occupational injury*” *OR* “*skin injury*” *OR* “*skin damage*”).	97
Science Direct	*COVID-19 AND* (“*protective devices*” *OR* “*personal protective equipment*”) *AND* (*skin OR* “*device related pressure injuries*” *OR* “*facial injuries*” *OR* “*occupational injury*” *OR* “*skin injury*” *OR* “*skin damage*”).	566
Web of Science	*COVID-19 AND* (“*protective devices*” *OR* “*personal protective equipment*”) *AND* (*skin OR* “*device related pressure injuries*” *OR* “*facial injuries*” *OR* “*occupational injury*” *OR* “*skin injury*” *OR* “*skin damage*”).	42
SciELO	*COVID-19 AND* (“*dispositivos de protección*” *OR* “*equipo de protección personal*”) *AND* (*piel OR* “*lesiones por presión relacionadas con el dispositivo*” *OR* “*lesiones faciales*” OR “*lesión ocupacional*” *OR* “*lesión cutánea*” *OR* “*daño cutáneo*”)	1
TOTAL		851

### Data collection

The search was carried out in January 2021 through the CAPES Journal Portal, through access to the Federated Academic Community (*Comunidade Acadêmica Federada*, CAFe), with selection of *Universidade Federal do Maranhão*(UFMA) higher education institution, searching the following databases: Medical Literature Analysis and Retrieval System Online (MEDLINE) via the National Library of Medicine (PubMed); Cumulative Index to Nursing and Allied Health (CINAHL); *Literatura Latino-Americana e do Caribe em Ciências da Saúde*(LILACS); SCOPUS; Science Direct and Web of Science and Scientific Electronic Library Online (SciELO).

To improve the quality of data collection, the Rayyan app was used, developed by the Qatar Computing Research Institute (QCRI)^12^, to assist in the process of organizing and selecting the studies, as well as removing duplicates. In addition to that, the search was performed by two researchers, independently and simultaneously, following a search protocol directed to the guiding question and eligibility criteria. The inclusion criteria established for the selection of studies were original articles that answered the guiding question, covering injuries and/or preventive measures, published in English, Spanish or Portuguese. 

### Instrument used to collect the information

For data collection, categorization and interpretation, an adapted instrument was used^13^ with the following items: title of the publication; author(s); year of publication; journal; objective; type of study; level of evidence; types of injuries and preventive measures. 

### Data analysis

The level of evidence of the articles included was classified as follows:

Level 1 - Experimental research designs: 1.a) Systematic review of randomized controlled trials; 1.b) Systematic review of randomized, controlled trials and other study designs; 1.c) Randomized controlled trial; 1.d) Controlled, randomized pseudo-trials;
Level 2 - Quasi-experiment designs: 2.a) Systematic review of quasi-experimental studies; 2.b) Systematic review of quasi-experiment and other less-evidence study designs; 2.c) Prospectively controlled quasi-experiment studies; 2.d) Pre- and post-test or retrospective historical controlled group studies;
Level 3 - Observational - analytical designs: 3.a) Systematic review of comparable cohort studies; 3.b) Systematic review of comparable cohorts and other lower evidence study designs; 3.c) Cohort study with a control group; 3.d) Case-control study; 3.e) Observational studies without a control group;
Level 4 - Observational - descriptive studies: 4.a) Systematic review of descriptive studies; 4.b) Cross-sectional study; 4.c) Case series; 4.d) Case study;
Level 5 - Experts’ opinion - Laboratory bench research: 5.a) Systematic review of expert opinion; 5.b) Experts’ consensus; 5.c) Laboratory bench research/expert’s opinion^14^.

In addition to that, an analysis of the methodological quality was carried out using the instruments proposed by the JBI^15^ that contain a checklist with questions for each type of study with the following answer options: Yes; No; Not applicable or Not clear. After categorization of the studies, the data were synthesized for the descriptive analysis according to year of publication, language, study locus, objective, type of study, level of evidence, types of skin lesions and preventive measures related to PPE use in health professionals. 

## Results

**Figure 2:**
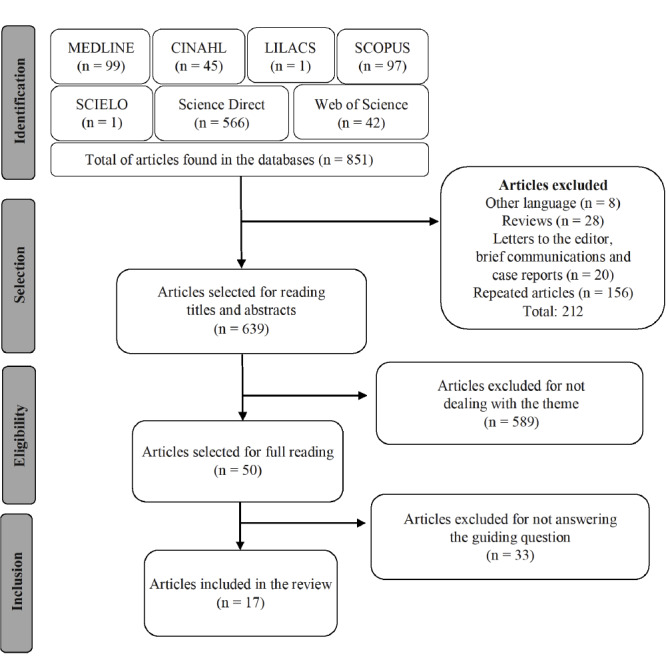
Flowchart corresponding to the selection of the articles included in the study. Imperatriz, MA, Brazil, 2021

Source: Adapted from PRISMA^16^.

A total of 851 articles were identified and, after analyzing the titles and abstracts and applying the inclusion and exclusion criteria, 50 articles were pre-selected for full reading. Among the 50 articles analyzed, 17 were included in the final sample of this review. The path followed to search and select the studies observed the PRISMA group recommendations^15^ and can be seen in the flowchart below (Figure 2).

The 17 articles that comprised the final sample were published in 2020 and 2021, in English and Spanish. The studies were carried out in the following countries: Bahrain, Brazil, China, India, Iraq, Mexico, Paris (France), Thailand, Turkey and the United States, with China (35.5%) being the country with the highest number of publications. The types of study were as follows: prospective cohort^17^ ^-^ ^18^ (11.8%), randomized clinical trial^19^ ^-^ ^20^ (11.8%) and descriptive cross-sectional^21^ ^-^ ^33^ (76.4%). When evaluating the level of evidence, 76.4% of the publications were classified as level 4b.

Figure 3 presents the characterization of the studies that were included in the final sample, considering objective, country where the study was carried out, language, level of evidence and methodological quality of the study.

**Figure 3:** Characterization of the primary studies included in the sample. Imperatriz, MA, Brazil, 2021

Authors	Objective	Language/Country	Level of Evidence	Methodological quality of the study
Smart, et al. (2020)^17^	To determine whether a silicone-based dressing worn under an N95 mask is safe and beneficial for preventing facial skin injury without compromising the mask’s seal.	English/Bahrain	3.c	Seven out of 11 points in the JBI* checklist for *Cohort studies*
Yildiz, et al. (2021)^18^	To determine the effect of the prophylactic dressing on preventing skin lesions due to PPE use in health professionals working with COVID-19 patients.	English/Turkey	3.c	Eight out of 11 points in the JBI* checklist for *Cohort studies*
Hua, et al. (2020)^19^	To analyze the short-term effects of N95 respirators and medical masks on the physiological properties of the skin and to report adverse skin reactions.	English/China	1.c	Eight out of 13 points in the JBI* checklist for *Randomized controlled trials*
Gasparino, et al. (2021)^20^	To compare the use of foam and extra-thin hydrocolloid dressing in the prevention of pressure ulcers associated with the use of PPE by health professionals working on the front line against the Coronavirus.	English/Brazil	1.c	Eight out of 13 points in the JBI* checklist for *Randomized controlled trials*
Jiang, et al. (2020)^21^	To investigate the prevalence and characteristics of pressure ulcers among health team members.	English/China	4.b	Six out of eight points in the JBI* checklist for *Analytical cross-sectional studies*
Hu, et al. (2020)^22^	To explore adverse skin reactions among health professionals who use PPE.	English/China	4.b	Six out of eight points in the JBI* checklist for *Analytical cross-sectional studies*
Shanshal, et al. (2020)^23^	To comprehensively examine the effect of COVID-19 on different aspects of the medical practice.	English/Iraq	4.b	Four out of eight points in the JBI* checklist for *Analytical cross-sectional studies*
Xia, et al. (2020)^24^	To identify physical and psychological effects of using PPE and its related safety measures on health care workers in Wuhan, China, in response to the pandemic.	English/China	4.b	Six out of eight points in the JBI* checklist for *Analytical cross-sectional studies*
Pacis, et al. (2020)^25^	To formulate procedures to protect the integrity of health care workers’ facial skin when caring for COVID-19 patients.	English/United States	4.b	Five out of eight points in the JBI* checklist for *Analytical cross-sectional studies*
Agarwal, et al. (2020)^26^	To identify the difficulties encountered by health professionals during PPE use and to propose ways and means to help them overcome such difficulties.	English/India	4.b	Four out of eight points in the JBI* checklist for *Analytical cross-sectional studies*
Yuan, et al. (2020)^27^	To understand the possible skin, respiratory, nervous and digestive reactions in Chinese health professionals who use PPE in the fight against COVID-19.	English/China	4.b	Six out of eight points in the JBI* checklist for *Analytical cross-sectional studies*
Jiang, et al. (2020)^28^	To investigate the prevalence, characteristics and preventive status of skin lesions caused by PPE in the health team.	English/China	4.b	Six out of eight points in the JBI* checklist for *Analytical cross-sectional studies*
Daye, et al. (2020)^29^	To evaluate the skin problems and dermatological quality of life of health professionals due to PPE use.	English/Turkey	4.b	Four out of eight points in the JBI* checklist for *Analytical cross-sectional studies*
Atay, et al. (2020)^30^	To examine the physical issues related to PPE and prolonged wear time experienced by nurses during the COVID-19 pandemic.	English/Turkey	4.b	Six out of eight points in the JBI* checklist for *Analytical cross-sectional studies*
Techasatian, et al. (2020)^31^	To analyze the prevalence and possible risk factors to prevent face mask-related skin reactions during COVID-19.	English/Thailand	4.b	Six out of eight points in the JBI* checklist for *Analytical cross-sectional studies*
Masen, et al. (2020)^32^	To provide a practical lubricating solution for COVID-19 front line health care workers working a four-hour shift or longer wearing PPE.	English/Paris (France)	4.b	Six out of eight points in the JBI* checklist for *Analytical cross-sectional studies*
Erize-Herrera, et al. (2020)^33^	To describe the frequency of cutaneous manifestations caused by PPE use in health professionals and the risk factors for developing them.	Spanish/Mexico	4.b	Four out of eight points in the JBI* checklist for *Analytical cross-sectional studies*

* In the checklist some answers were rated as Not Applicable or Not Clear.

Table 1 shows the types of injuries related to PPE use. The main PPE items associated with the occurrence of skin injuries were as follows: surgical or N95 mask, goggles, face shield, gloves and protective clothing. The most frequent injuries caused by mask use were the following pressure ulcer (stage 1)^17^ ^-^ ^18^ ^,^ ^21^ ^,^ ^23^ ^-^ ^24^ ^,^ ^28^ (35.2%); acne^22^ ^,^ ^29^ ^-^ ^30^ ^,^ ^32^ (23.5%); skin depression^17^ ^,^ ^19^ ^,^ ^22^ ^,^ ^27^ (23.5%); irritant contact dermatitis^22^ ^,^ ^29^ ^,^ ^32^ (17.6%); rash^23^ ^,^ ^26^ ^,^ ^30^ (17.6%); pressure ulcer (stage 2)^21^ ^,^ ^23^ (11.7%) and injuries associated with moisture^18^ ^,^ ^24^ (11.7%).

Regarding the injuries associated with using protective eyewear, stage 1 pressure ulcers stood out^18^ ^,^ ^23^ ^-^ ^24^ ^,^ ^28^(23.5%), followed by stage 2^21^ ^,^ ^23^ ^,^ ^28^ (17.6%). The injuries related to face shield use that showed higher frequency were stage 1 pressure ulcers^21^ ^,^ ^23^ ^-^ ^24^ ^,^ ^28^ (23.5%). Regarding the use of gloves, xerosis^21^ ^,^ ^23^ ^-^ ^24^ ^,^ ^28^ ^-^ ^29^ (23.5%) and fissures^22^ ^,^ ^29^ ^-^ ^30^ (17.6%) stood out. As for the injuries associated with using protective clothing, irritant contact dermatitis stood out^23^ ^,^ ^26^ (11.7%).

**Table 1:** Frequency of the types of injuries related to using a surgical or N95 mask, goggles, face shield, gloves and protective clothing according to the studies found. Imperatriz, MA, Brazil, 2021

Personal Protective Equipment	Types of injuries	Frequency n (%)*	Studies found
Surgical or N95 mask	Pressure ulcer (stage 1)^†^	6 (35.2)	17,18,21,23,24,28
	Acne	4 (23.5)	22,29,31,33
	Skin depression	4 (23.5)	17,19,22,27
	Irritant contact dermatitis	3 (17.6)	22,29,33
	Cutaneous rash	3 (17.6)	23,26,31
	Pressure ulcer (stage 2)^†^	2 (11.7)	21,23
	Injuries associated with moisture	2 (11.7)	18,24
	Maceration	1 (5.8)	33
	Cheilitis	1 (5.8)	30
	Pressure ulcer (stage 3)^†^	1 (5.8)	21
	Urticaria	1 (5.8)	22
	Pustule	1 (5.8)	23
	Papule	1 (5.8)	23
	Xerosis	1 (5.8)	33
	Bubbles	1 (5.8)	33
Safety goggles	Pressure ulcer (stage 1)^‡^	4 (23.5)	18,23,24,28
	Pressure ulcer (stage 2)^‡^	3 (17.6)	21,23,28
	Cutaneous rash	2 (11.7)	23,33
	Skin depression	1 (5.8)	27
	Pressure ulcer (stage 3)^‡^	1 (5.8)	21
	Papule	1 (5.8)	23
	Pustule	1 (5.8)	23
Face shield	Pressure ulcer (stage 1)^§^	4 (23.5)	21,23,24,28
	Pressure ulcer (stage 2)^§^	2 (11.7)	23,28
	Cutaneous rash	2 (11.7)	20,23
	Skin depression	1 (5.8)	27
	Injuries associated with moisture	1 (5.8)	18
	Folliculitis	1 (5.8)	18
	Friction injury	1 (5.8)	18
	Pressure ulcer (stage 3)^§^	1 (5.8)	21
	Papule	1 (5.8)	23
	Pustule	1 (5.8)	23
Gloves	Xerosis	4 (23.5)	22,30,31,33
	Fissures	3 (17.6)	22,29,30
	Flaking	2 (11.7)	29,33
	Cutaneous rash	2 (11.7)	22,26
	Irritant contact dermatitis	1 (5.8)	26
	Maceration	1 (5.8)	33
	Bubbles	1 (5.8)	33
	Worsening of preexisting dermatosis	1 (5.8)	29
	Friction injury	1 (5.8)	18
	Injuries associated with moisture	1 (5.8)	18
	Urticaria	1 (5.8)	22
	Lichenification	1 (5.8)	29
Protective clothing	Irritant contact dermatitis	2 (11.7)	23,26
	Cutaneous rashes	1 (5.8)	26
	Injuries associated with moisture	1 (5.8)	18
	Xerosis	1 (5.8)	22

*Total “n” value corresponding to the sample of 17 articles. The percentage exceeds 100% as there was more than one type of lesion in the same article included in the sample; ^†^Frontal, Nasal Bridge, Auricular and/or Mandibular; ^‡^Frontal, Nasal Bridge, Auricular; ^§^Frontal

The main preventive measures related to injuries caused by PPE use were grouped according to the type of PPE. For injuries resulting from using a mask, goggles and face shield, most of the articles^17^ ^-^ ^18^ ^,^ ^20^ ^,^ ^23^ ^,^ ^28^ (27.7%) recommended using a hydrocolloid or foam dressing in the pressure regions, but other recommendations were also mentioned, such as using moisturizers and emollients for protection, especially when the professionals are not treating patients, in addition to correct adjustment of the mask and to using a protective ear strap.

As recommendations for the use of gloves, the studies cited the following: washing and drying the hands and feet well; applying a barrier cream before and after using the PPE; avoiding wearing gloves for a long time; and using cotton gloves or a layer of plastic gloves inside the latex gloves. Regarding the use of protective clothing to avoid injuries, the recommendation is to apply a barrier cream before and after using PPE items to avoid skin peeling and drying (Figure 4).

**Figure 4:** Preventive measures for the occurrence of skin injuries related to the use of Personal Protective Equipment. Imperatriz, MA, Brazil, 2021

Recommendations for the prevention of injuries related to the use of masks, glasses and face shields
- Use an alcohol-free liquid protective barrier in areas of direct contact with PPE (e.g.: nose, cheeks, forehead, behind the ears) to protect the skin from moisture and friction. Before applying, make sure that the area is free of makeup. Do not apply to eyes or eyelids and allow to dry for 90 seconds before putting on the PPE^25^.
- Apply a thin hydrocolloid or foam dressing and adjust the material to the skin without tensioning it on the bridge of the nose, cheeks and forehead, covering the area where the mask, face shield and goggles rest^17^ ^-^ ^18^ ^,^ ^20^ ^,^ ^23^ ^,^ ^28^.
- Use acrylate-based moisturizers frequently, especially when not in direct patient care^17^.
- Fit the N95 mask correctly by fixing the clip and add a surgical mask for alignment^22^.
- Use a protective ear strap attached to the mask elastic^23^.
**Recommendations for the prevention of injuries related to the use of gloves and protective clothing**
- Wash hands with soap, detergent, soaps or light oils, without perfume and with the least number of preservatives, dry them well and apply talc powder to the hands and feet before putting on gloves and boots, in order to protect the skin from friction and excessive hydration. If the gloves are dry, do not put too much talc powder on them^22^.
- Apply specific hand cream regularly and avoid wearing gloves for a long time. Wearing cotton gloves or a layer of plastic gloves inside latex gloves helps protect against itching or irritation in people with a latex allergy^21^.
- Apply a barrier cream before and after using PPE to prevent skin flaking and dryness^28^ ^-^ ^29^.

In addition to the recommendations directed to each PPE category, some studies listed recommendations for general care measures in the prevention of injuries. They were as follows: PPE removal^26^ and not using emollients or moisturizing creams when the PPE is used for a period longer than four hours, as they can result in excessive shear forces on the skin and, in such cases, they must be replaced by talc, lanolin containing vaseline or a mixture of coconut oil, cocoa butter and beeswax, as they provide excellent long-lasting lubrication^32^.

## Discussion

The COVID-19 pandemic required prolonged PPE use not only by the team of health professionals, but also by the entire hospital staff. Therefore, the number of professional service providers who needed to use PPE increased considerably, as did the skin lesions associated with the intensive use of these materials. Given the lack of studies with in-depth and updated information on the occurrence of skin lesions associated with PPE use and prevention strategies, there was a growing concern in researchers to produce knowledge on the theme to assist the multiprofessional team^7^. 

In the studies identified in this review, surgical or N95 masks, goggles, face shields and gloves were the main factors responsible for the onset of injuries. A survey carried out in China identified that 95.1% of the health professionals who regularly used N95 masks more than 12 hours a day for a mean of 3.5 months complained of some type of skin involvement^22^. Another study observed that 47.3% of the interviewees, who used PPE items for more than four hours, also presented some type of impairment^28^. These data indicate that, in addition to the type of PPE, use time is also a risk factor for the onset of injuries. 

The WHO indicates that N95, PFF2 or equivalent masks should not be used for a period longer than four hours due to the risk of discomfort and pain. However, in times of a public health emergency and when there is shortage of equipment, they can be used, without removing them and without losing their protective effectiveness, for a long period, when caring for several patients who have the same diagnosis^34^.

Persistent PPE use over the recommended time requires measures to avoid or minimize the occurrence of injuries. However, this recommendation was not observed in a study carried out in China, as only 17.7% of the interviewees used prophylactic dressings or lotions on the skin during the assistance provided. The authors believe that the fact that 42.8% of the evaluated professionals had skin lesions is related to the lack of training or to the fear of using dressings for protection, which prevented correct fixation of the PPE^28^.

Among the types of skin injuries, stage 1 pressure ulcers were among the most frequent in those who used PPE items. In China, a study identified pressure ulcers in 30% of the professionals interviewed and, of these, 81.1% were in stage 1 and 18.3% were in stage 2^28^. Another study identified that 25.58% of the health professionals presented pressure ulcers on the face^35^, while another study evidenced that 68.9% had pressure ulcers on the nasal bridge associated with prolonged PPE use^22^.

Such lesions are formed from a sustained mechanical load that is applied to soft tissues near a bony prominence. It is noted that magnitude of the load depends on the time it is used to cause harms; therefore, a low load applied for a prolonged period causes as much tissue damage as a high load for a short period^36^. In the case of health professionals working on the front lines against COVID-19 and in an attempt to avoid respiratory infection by the Coronavirus, it was common for them to adjust their N95 masks so that the edge was in excessively close contact with the skin and the metal clip fitted firmly over the nose to ensure a complete seal^22^. This fact may have contributed to the formation of lesions on the face and nasal bridge of these professionals.

A study that evaluated the protective measures for skin lesions found that mild emollients, silicone cream and film dressing applied in the region of greater mechanical pressure between the mask and the skin were more accepted to the detriment of using foam dressings, as these require greater skill to apply and adhere them correctly. However, the authors highlighted that using creams and emollients in large amounts can facilitate adhesion of dirt, making it difficult to reuse the mask in a context of scarcity, while the film dressing needs to be removed carefully to avoid pain^37^. 

Foam dressings with a silicone edge are beneficial when used correctly, as shown in a study developed by nurses in Bahrain. The professionals who used the dressing presented fewer skin reactions after one hour of continuous use when compared to those who did not use any protection. Before using the face mask, it is recommended to properly prepare and cut the foam dressing and place it on the nose, on the side of the face, below the chin, central region of the forehead and close to the ears, as well as use moisturizers after removal^17^. A number of research studies have shown that the dressing does not interfere with sealing of the masks^17^ ^,^ ^37^.

Facial acne was also among the most frequent types of lesions in the studies analyzed. A survey carried out in Singapore with professionals who were assisting in the fight against the Severe Acute Respiratory Syndrome (SARS) infection in 2003 identified acne as the main skin reaction (65%) associated with prolonged use of N95 masks^38^. Another research study carried out in India pointed to acne as the fifth most common dermatosis among health professionals who used PPE items for more than eight hours a day, behind irritant contact dermatitis, allergic dermatitis, pressure and friction marks and sweat dermatitis^35^. 

The onset of acne can be explained by the obstruction of the pilosebaceous ducts caused by prolonged occlusion and local pressure on the skin due to using the mask which, together with mental stress, extremely heavy workload and sleep deprivation, contributes to the emergence of acne or to aggravation of the existing problem. For acne control, in addition to general skin care, it is recommended to wash the face twice a day, choose suitable facial cleansers, use light cosmetics or avoid them and, in mild to severe cases, use an appropriate drug treatment with topical antibiotics and/or retinoid ointment to systemic treatment, with monocycline or isotretinoin, as directed by a physician^39^.

Another type of skin lesion that stood out in the studies included in this review was skin depression, which can occur due to prolonged use of masks, glasses and face shields^19^ ^,^ ^22^ ^,^ ^27^. This type of lesion usually regresses spontaneously^22^ ^,^ ^27^; however, a recommended treatment for redness and swelling consists of using a hydropathic compress with three to four layers of gauze soaked in cold water or saline every two hours, followed by the application of moisturizers^22^. Prevention of this type of injury, in addition to those already mentioned, is about the correct use of facial PPE, with the proper size, well-fitted and not tight^18^ ^,^ ^40^ ^-^ ^41^ and using N95 mask straps over the head^41^, in addition to using cotton disks fixed with hypoallergenic surgical tape in the nasal and zygomatic region to avoid friction^42^.

The presence of dermatitis was identified in health professionals who used PPE items, mainly due to using masks and alcohol gel^22^ ^,^ ^43^ ^-^ ^44^. Such dermatitis cases can be divided into two types: allergic contact dermatitis and irritant contact dermatitis. The latter comprises a non-immunological response that commonly affects the hands and face and occurs as a result of direct damage to the skin by chemical or physical agents in a way that the skin cannot repair itself quickly. Allergic contact dermatitis is a delayed type IV hypersensitivity reaction to an external allergen that occurs only in an individual who has been previously sensitized. This re-exposure to the allergen results in circulating memory T cells that travel to the skin and trigger an immune reaction that causes skin inflammation, typically within 48 hours^45^.

Generally, the exposure time to allergen content and/or moisture has been identified as the main risk factor for contact dermatitis; therefore, wearing a mask and glasses for more than six hours or washing the hands more than ten times a day can increase the risk of local dermatitis. It is also important to consider additional exposure to the mask’s material, as well as repeated hand washing outside the work environment as a way to prevent SARS-CoV-2 infection^44^. 

Preventive measures for contact dermatitis include the application of emollients before using masks and not using them with the material responsible for burning and itching. However, if this is not possible, it is recommended to use preventive covers and/or dressings that prevent the mask from coming into direct contact with the skin, in addition to extra care such as avoiding using 75% ethanol for skin cleaning and using too hot water. Mild cutaneous rashes may spontaneously improve after three to five days without further treatment^43^. In turn, aggravation of pre-existing conditions such as psoriasis, atopic dermatitis and allergic reactions may require more complex management, including temporary distancing of these health professionals from work^44^.

Regarding the use of gloves and protective clothing, injuries such as dry skin, itching and cutaneous rashes were present in professionals who used these PPE items for more than ten hours a day for a mean of 3.5 months^22^. Xerosis was identified in a Mexican survey as one of the main lesions found in health professionals (90.35%), followed by desquamation, erythema, fissures, vesicles and maceration, whose main affected area was the hands. The data obtained showed a significant association between lesions and using alcohol gel, with a 1.81-fold increase in the risk of xerosis^33^. This lesion is characterized by a reduction in the amount and/or quality of lipids and hydrophilic substances on the skin. Management of xerosis is based on the regular use of a combination of topical substances that are intended to improve hydration, compensate for the lack of lipids and improve the skin barrier function^46^.

A research study, which evaluated the main skin lesions and their risk factors in the COVID-19 pandemic context in the health team, found that maceration was present in 52.9% of the sample^47^. This type of injury results from prolonged exposure to moisture, which causes the skin to soften and break down so that the conjunctival fiber pulls away and the skin often appears white. Generally, this injury can be prevented by using semipermeable materials, which allow water vapor to escape, and the indicated treatment consists of using superabsorbent dressings to reduce excess moisture^48^. 

With regard, specifically, to PPE use by health professionals, other preventive measures are recommended, such as choosing the appropriate size of gloves and rubber boots, applying ostomy powder on hands and feet, waiting, after hand washing and/or using alcohol gel on the skin, that it dries completely before using PPE items, as well as frequently exchanging these materials. These measures help protect the skin against friction and excessive hydration. In the case of persistent maceration, using an astringent cream, such as zinc oxide, helps to treat this type of lesion^43^. 

Adverse skin reactions due to using coats and protective clothing are less common; however, their occurrence can be associated with the moisture generated by heavy fabrics and with the need to use them for long periods of time. Strategies such as removing the clothes for a few minutes or changing them frequently can help reduce those reactions^22^.

A paper developed by nurses in Bahrain used a mnemonic with the word HELP to help health professionals incorporate skin care before, during and after using PPE items. In general, it was recommended to drink water regularly, keep the skin clean and hydrated (especially hands and face), observe the PPE use time, maintain a balanced diet of calories and proteins, use barrier creams ten minutes before using PPE items and protective coverings in the areas of greatest skin contact with them^17^. 

Despite the growing number of publications on the prevalence of injuries, prevention and treatment guidelines, studies that used a methodological approach that provided high levels of scientific evidence are still incipient. As this is an emerging topic from a new disease, the lack of a common term, descriptors or specific keywords for this, which may have left some study out the research result, stands out as a limitation of this study. In addition to that, the low number of randomized clinical trials precludes generalization of the results.

The results obtained can help health professionals, especially nurses, to know the types of skin lesions frequently associated with PPE use and which measures can be taken to prevent their occurrence. And also to support managers in carrying out training sessions for the prevention of possible injuries and incorporation of recommendations into the institution’s routine.

## Conclusion

Skin lesions caused by PPE use in health professionals were more notorious during the COVID-19 pandemic, when it was possible to observe the consequences of their prolonged use on a larger scale. Most of the professionals who worked in direct care for patients infected with the new Coronavirus presented some type of skin impairment, especially those who used PPE items for a daily period of more than six hours. Masks, especially those of the N95 type, were the main factor responsible for the occurrence of skin injuries, followed by goggles, face shields and gloves. As for the injury types, acne, pressure ulcers (stages 1 and 2), skin depression and xerosis were more frequently observed in the professionals.

The main measures to prevent skin damage involve routine care, such as keeping the skin hydrated, consumption of liquids and using moisturizers and emollients, instituting the habit of skin hygiene and avoiding using excessive makeup, in addition to using protective pads on bony prominences of the face, preferably with foam and silicone edges to facilitate removal, use properly adjusted PPE items and, if possible, avoid continuous use of PPE items for prolonged periods of time.
